# Upregulated epithelial junction expression represents a novel parameter of the epithelial radiation response to fractionated irradiation in oral mucosa

**DOI:** 10.1007/s00066-018-1302-6

**Published:** 2018-04-19

**Authors:** Sylvia Gruber, Nilsu Cini, Lisa-Marie Kowald, Julia Mayer, Andreas Rohorzka, Peter Kuess, Wolfgang Dörr

**Affiliations:** 1 Dept. Radiation Oncology/CD Lab. Med. Radiation Research for Radiation Oncology, Applied and Translational Radiobiology, Medical University/AKH Vienna, Währinger Gürtel 18–20, 1090 Vienna, Austria; 2 Department of Radiation Oncology, Kartal Dr. Lutfi Kırdar Training and Research Hospital, Health Science University, Istanbul, Turkey

**Keywords:** Fractionated irradiation, Oral mucositis, Adherens junctions, Tight junctions, Mouse model, Fraktionierte Bestrahlung, Orale Mukositis, Adherens Junctions, Tight Junctions, Mausmodell

## Abstract

**Purpose:**

During head and neck cancer treatment, the radiation response of the oral mucosa represents a frequent early side effect. Besides radiation-induced inhibition of proliferation, various other cellular responses occur. The radiation response of adherens and tight junction proteins was so far mostly investigated with large single-dose irradiation protocols, in vivo and in vitro. Therefore, the current study was initiated to investigate the impact of daily fractionated irradiation on the expression of adherens and tight junction proteins in vivo.

**Materials and methods:**

Fractionation with 5 × 3 Gy/week (days 0–4, 7–11) was given to the snouts of mice. Groups of 5 animals per day were euthanized every second day between day 0 (unirradiated controls) and day 14, and their tongues subjected to histological processing. Adherens junction marker (β-catenin and E‑cadherin) and tight junction marker (claudin-1 and occludin) expression was analysed in the oral mucosa of unirradiated controls and during two weeks of fractionated irradiation.

**Results:**

Adherens as well as tight junction marker proteins were rapidly and consistently upregulated in both the germinal as well as the functional layer of the oral mucosa. This represents a previously unknown parameter of the epithelial radiation response to clinically relevant fractionation protocols.

**Conclusion:**

Fractionated irradiation significantly enhanced the expression of all proteins investigated. This study revealed a new parameter of the epithelial radiation response to fractionated irradiation.

**Electronic supplementary material:**

The online version of this article (10.1007/s00066-018-1302-6) contains supplementary material, which is available to authorized users.

## Zusammenfassung

**Zielsetzung:**

Während der Behandlung von Kopf-Hals-Tumoren repräsentiert die Strahlenreaktion der Mundschleimhaut eine häufige und frühe Nebenwirkung. Abgesehen von strahleninduzierter Inhibierung der Zellteilung finden zahlreiche andere zelluläre Reaktionen statt. Die Strahlenreaktion von Zellverbindungsproteinen wurde bisher größten Teils – sowohl in vivo als auch in vitro – mit großen Einzeldosisprotokollen untersucht. Daher wurde die vorliegende Studie initiiert, um den Einfluss täglicher fraktionierter Bestrahlung auf die Expression der Zellverbindungsproteine in vivo zu untersuchen.

**Material und Methoden:**

Täglich fraktionierte Bestrahlung mit 5 × 3 Gy/Woche (Tage 0–4, 7–11) wurde auf die Schnauzen der Mäuse appliziert. Gruppen von 5 Mäusen pro Tag wurden jeden zweiten Tag zwischen Tag 0 und Tag 14 euthanasiert und ihre Zungen histologische aufbereitet. Die Expression von Markern für Adherens Junctions (β-Catenin und E‑Cadherin) sowie für Tight Junctions (Claudin-1 und Occludin) wurde in der Mundschleimhaut von nichtbestrahlten Kontrollen und über einen Fraktionierungszeitraum von 2 Wochen analysiert.

**Ergebnisse:**

Adherens- als auch Tight-Junction-Marker wurden sowohl in der germinativen als auch der funktionellen Schicht der oralen Schleimhaut rapide und beständig hochreguliert. Dies repräsentiert einen bisher unbekannten Parameter der epithelialen Strahlenreaktion auf klinisch relevante Fraktionierungsprotokolle.

**Schlussfolgerung:**

Fraktionierte Bestrahlung steigerte signifikant die Expression aller untersuchten Proteine. Diese Studie offenbarte einen neuen Parameter der epithelialen Strahlenreaktion nach fraktionierter Bestrahlung.

## Introduction

The epithelial radiation response often represents a dose-limiting early side effect, experienced by the majority of head and neck cancer (HNC) patients as confluent oral mucositis [1–3]. Oral mucositis is associated with severe pain, increased risk for local and systemic infections, and can necessitate unplanned treatment interruptions, thus negatively influencing tumour control probability. It has a major impact on the patient’s quality of life. Hospitalizations for supportive care are an important socio-economic factor [4]. Despite its clinical relevance, no biology-based treatment strategy has been implemented into clinical routine so far [5]. This can be partly attributed to the fact that the exact molecular radiopathological mechanisms underlying oral mucositis are not yet fully understood.

Oral mucosa is a typical turnover tissue, with a precise equilibrium of continuous proliferation in the germinal tissue compartments and cell loss at the surface. Radiation abolishes epithelial proliferation but does not influence the rate of superficial cell shedding. This leads to epithelial hypoplasia and subsequently denudation, which represents the primary cause of oral mucositis [6]. Importantly, the radiation-induced epithelial hypoplasia leads to a loss of the tissue barrier function [7]. Mucosae such as the oral epithelium form a barrier against potentially harmful chemicals and microbiota [8]. Junctional complexes are critically involved in the integrity of the mucosal barrier [9]. They are composed of transmembrane proteins, which interact with multiple intracellular adaptor proteins. Tight junctions represent intercellular gates; their size and charge selectively regulate the transport of small molecules across the paracellular space. The main barrier function-related components of tight junctions are the proteins claudin and occludin. Adherens junctions act as paracellular anchors, regulating cell–cell and cell–matrix adhesion; thus, they are responsible for the integrity of the epithelial barrier. The main transmembrane component of the adherens junction complex is E‑cadherin, which together with β‑catenin and α‑catenin forms a tertiary complex that binds to the actin cytoskeleton in a force-dependant manner [10–12]. Other than maintaining a polarized epithelial phenotype and stabilizing cell–cell interaction, β‑catenin also functions as a key regulator of the canonical Wnt signalling network. Upon Wnt ligand stimulation, cytoplasmic β‑catenin translocates to the nucleus and activates transcription factor-dependent expression of genes involved in embryonic development, tissue morphogenesis, cellular adhesion, and tumourigenesis [13–15]. Selectivity between adhesion or transcription is mutually exclusive and depends mainly on the structural configuration of β‑catenin [16, 17]. E‑cadherin appears to influence Wnt-dependent gene expression only via the availability of β‑catenin. Knockout of E‑cadherin in a cell line with active Wnt resulted in junctional disruption and augmented β‑catenin-dependent transcriptional activity, without, however, change in the transcriptional program. No effects were found when the E‑cadherin knockdown was introduced in a cell line that had no active Wnt signalling, hence loss of E‑cadherin alone appears not to activate the Wnt/β-catenin signalling axis [18]. A broad array of genes are activated via the Wnt/β-catenin pathway, including tight and adherens junction complex components. The tight junction proteins claudin and to a lesser extent occludin were shown to play pivotal roles in regulating the epithelial microenvironment, including proliferation [19]. Aberrant expression of both proteins in the membrane was associated with multiple malignancies and their clinicopathological significance, including oral squamous cell carcinoma [20–23]. In this study, the expression of tight junction markers (claudin-1, occludin) as well as adherens junction proteins (E-cadherin and β‑catenin) was analysed over the course of 2 weeks of clinically relevant fractionated irradiation.

## Methods

All experiments were performed according to the current animal welfare legislation with approval by the respective authorities (file no. BMWF 66.009/0039-II/3b/2014).

### Animals and housing

For all experiments, mice of the inbred C3H/Neu strain from the breeding colony of the Department of Biomedical Research, MedUni Vienna, were used. Mice of both genders were included in the experiments, since earlier studies excluded gender effects on the mucosal radiation response [24]. Mice were housed in a conventional husbandry under controlled temperature (22 ± 2 °C) and humidity (55 ± 10%) and a day/night rhythm of 12 h. The animals were housed in Makrolon® cages, 1284L Eurostandard Type II L with a floor area of 530 cm^2^ (Techniplast GmbH, Hohenpeißenberg, Germany), maximum 5 animals per cage, on aspen wood bedding (ABEDD-LAB & VET Service GmbH, Vienna, Austria). The mice had free access to standard maintenance diet (ssniff Spezialdiäten GmbH, Soest, Germany) and fresh water from standard Perspex drinking bottles ad libitum. The age of the mice at the onset of the experiments ranged from 8 to 12 weeks.

### Experimental design

Over the course of 14 days, animals received fractionated irradiation with 5 × 3 Gy per week. Irradiation was administered daily between 10 and 12 am over a course of 2 weeks, Mondays to Fridays (days 0–4 and 7–11). On the weekends (days 5–6 and 12–13), no irradiation was given to the animals, similar to conventional patient protocols.

In 2‑day intervals, groups of animals (*n* = 5) were sacrificed and their tongues excised at the base for analyses of irradiation-induced epithelial marker expression changes. Five unirradiated mice served as a control group.

### Irradiation technique

For all irradiation procedures, a YXLON Maxishot X‑ray unit (Yxlon International X‑ray GmbH, Hamburg, Germany) was used. Dosimetric commissioning was performed for all used set-ups [25]. Standard dosimetric quality assurance was performed regularly and the dose rate was found to be constant. Adjustment of the irradiation time thus defined the delivered dose.

Fractionated irradiation with 3 Gy per day was given to the whole snouts of the animals. Un-anaesthetized animals were guided into a set-up of plastic tubes (inner diameter 2 cm). The snouts were positioned in conical holes (10 mm → 6 mm) of a Perspex block at the front end of the tubes. The rear ends were closed to prevent withdrawal of the animals. The bodies of the mice were shielded caudally from a plane from the eyes to the throat with 12 mm of the Pb-Bi-Sn alloy MCP-96. The treatment volume thus included the snouts with the entire tongue. The set-up for simultaneous irradiation of 8 animals was positioned in a standardized way in the central beam of the irradiation device. For fractionated irradiation, the YXLON Maxishot device was operated at 200 kV with a tube current of 20 mA and a focus size of 5.5 mm. An additional 4 mm Al and 0.6 mm Cu beam filter was used, which resulted in a dose rate of ca. 1 Gy/min at the focus-to-skin distance of 45.5 cm. The dose homogeneity between the individual snout positions was 3.2 ± 0.5%. The beam direction was vertical.

### Immunohistochemistry

Histological preparation procedures have been reported in detail previously [26]. Stainings comprised 30 min incubation at 95 °C, dewaxing with Leica Bond Dewax solution (Leica Biosystems, Inc., Buffoalo Groove, IL; Cat no. AR9222), antigen retrieval with Bond Epitope Retrieval 1 solution (Leica Biosystems, Inc., Buffoalo Groove, IL; Cat no. AR9961) and blocking of unspecific binding sites with 2% goat serum. Primary antibody binding was visualized with diaminobenzidine chromogen and a haematoxylin counterstain, using the Leica Bond Refine Detection kit (Leica Biosystems, Inc., Buffoalo Groove, IL; Cat no. DS9800). Primary antibodies were diluted in the Leica Bond Antibody Diluent buffer (Leica Biosystems, Inc., Buffoalo Groove, IL; USA, Cat no. AR9352) as follows: anti-claudin-1 1:200 (Abcam, Cambridge, MA, USA; Cat no. 15098; rabbit polyclonal), anti-occludin 1:200 (Abcam, Cambridge, MA, USA; Cat no. 222691; rabbit polyclonal), anti-E-cadherin 1:3000 (Abcam, Cambridge, MA, USA; Cat no. 76055; rabbit polyclonal) and anti-β-catenin 1:700 (Abcam, Cambridge, MA, USA; Cat no. 2365; rabbit polyclonal).

### Histological analysis

Microscopic analyses were performed field by field with an Axio Lab.A1 HAL 35 (Carl Zeiss Microscopy, LLC, Thornwood, NY, USA) at 400× magnification. The lower mouse tongue epithelium was analysed from the tip to the end of the tongue. A minimum of five visual fields were evaluated. In each visual field the number of marker-positive cells was normalized to the total number of cells, visualized by haematoxylin nuclear staining. The fraction of marker-positive cells was evaluated separately for the germinal and the functional epithelial layer. Additionally, the staining intensity, corresponding to the amount of protein expressed, was assessed semi-quantitatively with an arbitrary score from 0 (no signal), 1 (weak signal), 2 (intermediate signal) to 3 (strong signal). Staining intensity was scored per visual field, not for each marker-positive cell individually. Staining homogeneity was good. Marker-positive cells and their respective staining intensity were evaluated by two independent and experienced researchers in a blinded fashion after extensive training. Intra-observer variability was found to be negligible. Inter-observer variability was low and results in good agreement.

### Statistical analysis

For statistical analysis and graphical representation, the SPSS statistical software (SPSS Inc., Chicago, IL, USA) and GraphPad Prism 5 (GraphPad Software, Inc., CA, USA), respectively, were used. Mean values and standard deviation (SD) were calculated for each experimental group. The analysis of variance (one-way ANOVA) was used to test for the significance of a difference between the mean values. A *p*-value of <0.05 was regarded as statistically significant.

## Results

**Fig. 1:**
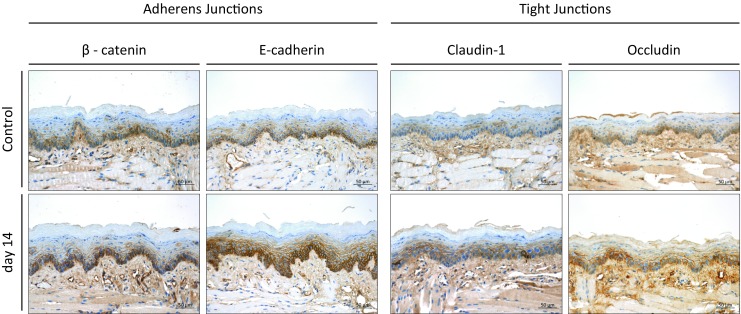
Augmentation of adherens junction marker (β-catenin and E‑cadherin) and tight junction marker (claudin-1 and occludin) expression during daily fractionated irradiation. Figures illustrate marker expression in unirradiated controls and on day 14 after fractionated irradiation with 10 × 3 Gy on days 0–4 and 7–11. All proteins increased significantly in both epithelial layers during the irradiation treatment. Scale bar: 50 µm

Representative histophotographs of immunohistochemical staining for β‑catenin, E‑cadherin, claudin-1 and occludin in untreated control mucosa and on day 14 after 10 × 3 Gy are presented in Fig. 1.

### Adherens junctions—β-catenin and E‑cadherin

#### Germinal layer

**Fig. 2:**
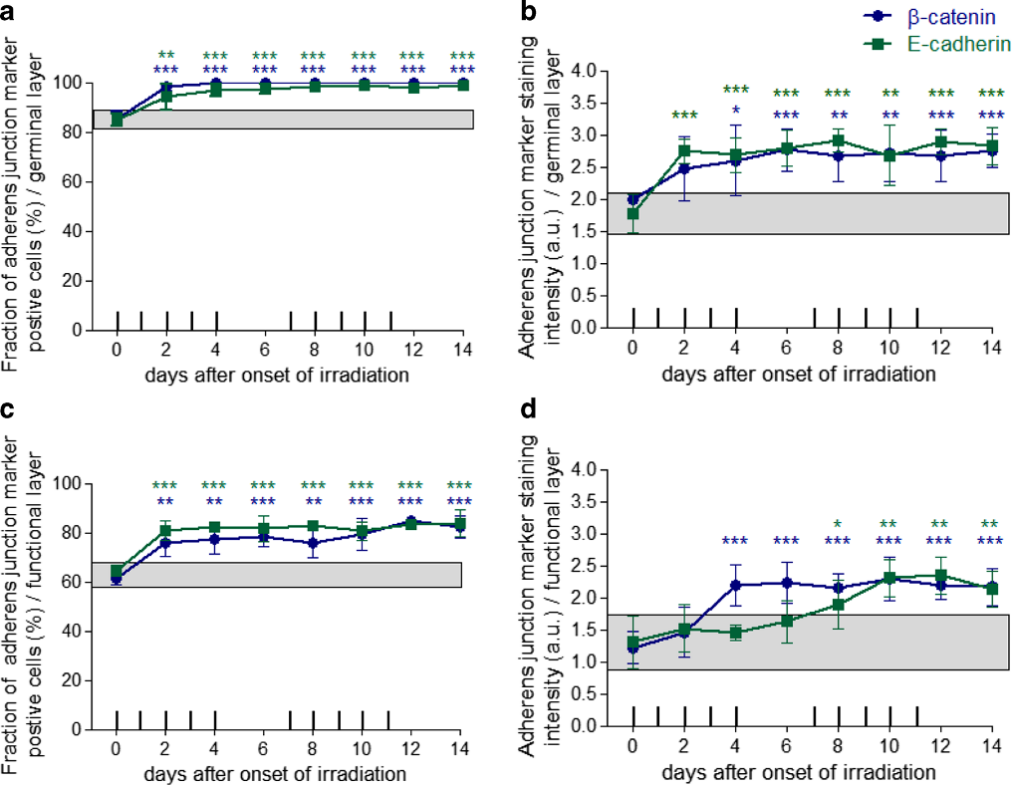
Effect of daily fractionated irradiation on adherens junction marker expression. The relative number of β‑catenin- and E‑cadherin-positive epithelial cells and their respective staining intensity were analysed, in the germinal (**a,** **b**) and functional compartments (**c,** **d**), respectively. The staining signal intensity was scored semi-quantitatively with an arbitrary score of 0 (no signal) to a maximum of 3 (strong). Adherens junction markers were analysed in unirradiated control samples and over the course of 14 days of fractionation with a daily dose of 3 Gy*. Data points* represent the mean of 5 animals, *error bars* indicate ±1 standard deviation (SD). The *shaded areas* illustrate the mean (±1 SD) from 5 control animals. The fractionation protocol is indicated on top of the abscissae; ^*^p ≤ 0.05, ^**^p ≤ 0.01, ^***^p ≤ 0.001

In control epithelia, approximately 85% of the germinal cells were positive for β‑catenin and E‑cadherin. With the onset of fractionation, the expression of both adherens junction marker proteins increased to levels between 94 and 98% within 2 days. A significant increase compared to the control values was found on all days investigated. From day 2 onwards, β‑catenin expression levels increased to 98% (*p* ≤ 0.001 for all days investigated). E‑cadherin expression increased from 85 to 94% on day 2 (*p* = 0.007) and further to values between 96% on day 4 and 99% on day 14, resulting in *p* ≤ 0.001 from day 4 onwards (Fig. 2a).

The β‑catenin staining intensity increased from 2 a. u. in control specimen to a maximum of 2.7 a. u. on day 14 (*p* ≤ 0.001). Likewise, E‑cadherin staining intensity increased from 1.8 a. u. in untreated samples to values between 2.7 a. u. on day 2 and 2.9 a. u. on day 14 (*p* ≤ 0.001 for days 2–14; Fig. 2b).

#### Functional layer

In the functional tissue layer, β‑catenin and E‑cadherin were expressed by 62–65% of cells in unirradiated control specimen. During radiotherapy, the expression of both adherens junction markers increased to 85% until day 14. Compared to control epithelia, significantly increased β‑catenin expression was found on day 2 (*p* = 0.002), day 4 (*p* = 0.002), day 6 (*p* ≤ 0.001), day 8 (*p* = 0.004) and from day 10 to day 14 with *p* ≤ 0.000. E‑cadherin expression was significantly potentiated (*p* ≤ 0.001) on days investigated (Fig. 2c).

The staining intensity for both adherens junction markers was 1.3 a. u. in control epithelia. With the onset of fractionation, the staining intensity of both proteins gradually increased. Significant staining intensity differences were found on all days investigated for β‑catenin (*p* ≤ 0.001) and on days 8 (*p* = 0.047), day 10 (*p* = 0.003), day 12 (*p* = 0.002) and day 14 (*p* = 0.008) for E‑cadherin (Fig. 2c).

### Tight junctions—claudin-1 and occludin

#### Germinal layer

**Fig. 3:**
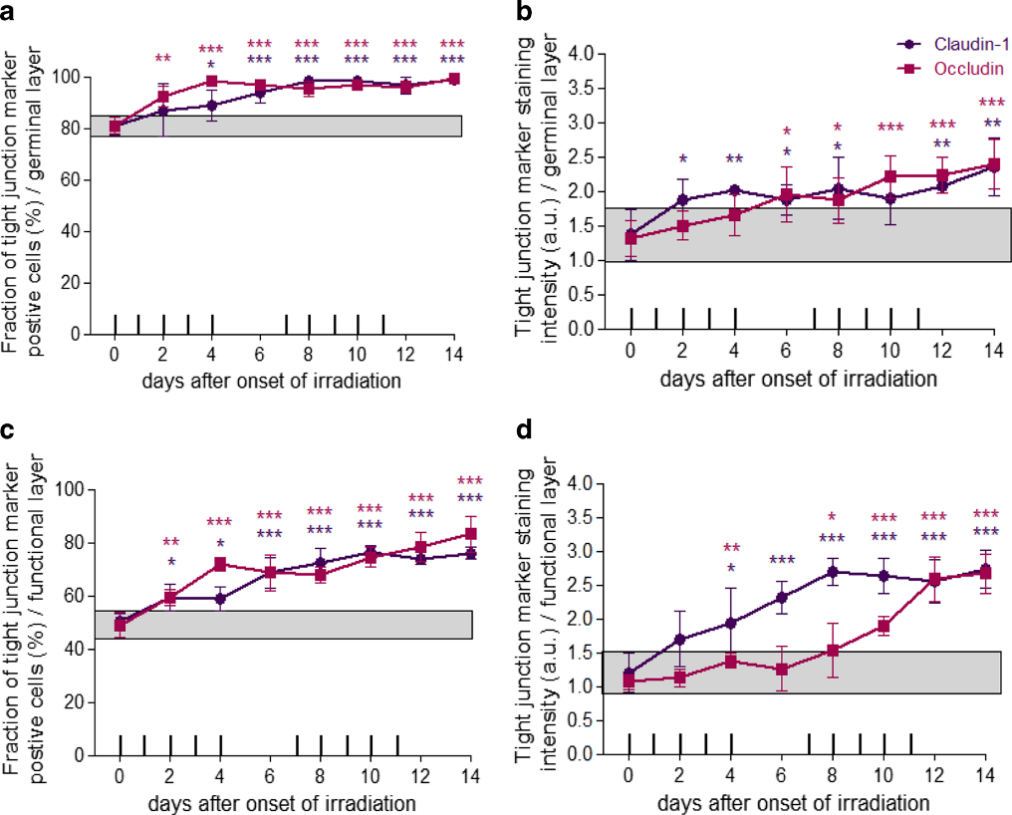
Effect of daily fractionated irradiation on tight junction marker expression. The relative number of claudin-1- and occludin-positive epithelial cells and their respective staining intensity were analysed, in the germinal (**a,** **b**) and functional compartments (**c,** **d**), respectively. The staining signal intensity was scored semi-quantitatively with an arbitrary score of 0 (no signal) to a maximum of 3 (strong). Adherens junction markers were analysed in unirradiated control samples and over the course of 14 days of fractionation with a daily dose of 3 Gy. *Data points* represent the mean of 5 animals, *error bars* indicate ±1 standard deviation (SD). The *shaded areas* illustrate the mean (±1 SD) from 5 control animals. The fractionation protocol is indicated on top of the abscissae; ^*^p ≤ 0.05, ^**^p ≤ 0.01, ^***^p ≤ 0.001

In control specimen, 81% of the germinal epithelial cells expressed claudin-1 and occludin. With the onset of fractionation, the percentage increased gradually for both proteins. At the end of the study period, on day 14, a maximum of 99% of claudin-1-and occludin-positive cells were observed in the germinal epithelial compartment. Significant claudin-1 expression differences as compared to unirradiated epithelia occurred on day 4 (*p* = 0.038) and from day 6 with *p* ≤ 0.001 until the end of the study period. Occludin expression was significantly upregulated from day 2 onwards (*p* = 0.002). From day 4 until day 14, occludin expression was observed in 95–99% of the germinal layer (*p* ≤ 0.001; Fig. 3a).

A gradual increase in the staining intensity was noted for both tight junction markers during the irradiation treatment. From 1.3 a. u. in unirradiated specimen, the staining intensity for both claudin-1 and occludin increased to a maximum of 2.4 a. u. on day 14. Significant differences were noted on day 2 (*p* = 0.048), day 4 (*p* = 0.005), day 6 (*p* = 0.037), day 8 (*p* = 0.033), day 12 (0.004) and day 14 (*p* = 0.005) for claudin-1. For occludin, significant differences were found from day 6 onwards with *p* = 0.017 on day 6, *p* = 0.02 on day 8 and *p* = 0.001 on days 10 to 14 (Fig. 3b).

#### Functional layer

In the functional compartment, 48% (occludin)–50% (claudin-1) of cells expressed the tight junction markers. During fractionation, the expression of both markers gradually increased until an expression maximum was observed for both proteins at the end of the study period. On day 14, 76% of functional epithelial cells expressed claudin-1 and 83% were occludin positive. Significantly increased claudin-1 expression was observed on all days (day 2: *p* = 0.014, day 4: *p* = 0.011, days 6–14: *p* ≤ 0.001). Occludin expression was significantly enhanced on all days throughout the study period with *p* = 0.005 on day 2 and *p* ≤ 0.001 from day 4 (Fig. 3c).

Before the onset of irradiation, the staining intensity of both markers was relatively weak, with 1.2 a. u. for claudin-1 and 1.1 a. u. for occludin. Daily irradiation potentiated the staining intensity, corresponding to the amount of protein expressed per positive cell, substantially during the study period. A maximum of 2.7 a. u. was observed for both proteins at the end of the study period on day 14, with significant differences compared to control specimen for claudin-1 on days 4 (*p* = 0.031) to 14 (*p* ≤ 0.001) and for occludin on day 4 (*p* = 0.009) and day 8 (*p* = 0.033) and from day 10 to day 14 from day 10 to day 14 with *p* ≤ 0.001 (Fig. 3d).

## Discussion

Severe (early) normal tissue side effects occur frequently during curative radio(chemo)therapy. Although accepted for the benefit of an optimal tumour treatment, early normal tissue side effects are associated with substantially reduced quality of life [3]. Hence, molecular changes leading to the development of a normal tissue response are of significant interest. Key mediators identified during the development of side effects can offer new targets for a biologically optimized treatment strategy [27–29].

This study was initiated to characterize the role of epithelial junctions during the development of oral mucositis, which is the most frequently occurring early side effect during radio(chemo)therapy of head and neck tumours. Primarily, oral mucositis manifests as a response to radiation-induced inhibition of epithelial proliferation. As the physiological superficial cell loss in the epithelium, characteristic for turnover tissues, continues independent of the treatment, epithelial hypoplasia and denudation, i. e. ulcerative lesions, develop. These are associated with a breakdown of the epithelial barrier function and therefore an increased risk for local and systemic infections [30, 31]. Epithelial junction proteins play a pivotal role in the maintenance and integrity of the tissue barrier function. So far, data on the radiation response of epithelial junctions are limited. De Carvalho and colleagues investigated the response of colon adenocarcinoma cells to irradiation and observed decreased transepithelial electrical resistance and disrupted junctional proteins after 2, 5 and 10 Gy. Notably, these effects were found to be reversible after 2 Gy, but persistent after higher doses [32]. Chai at al. report on structural alterations and tight junction protein degradation in mouse jejunum after single-dose whole-body irradiation with 6 Gy [33]. Similarly, Dublineau and colleagues concluded on epithelial disorganization characterized by tight junction disruption and accompanied by a loss of intestinal barrier function after irradiation with single doses between 6 and 12 Gy [34]. These findings were confirmed by Shukal et al., who reported on the radiosensitivity of junction proteins in a mouse model of gastrointestinal mucositis. A rapid disruption of occludin and claudin-1, E‑cadherin and β‑catenin occurred after total-body irradiation of 4 Gy [35]. Also, Thiagarajah et al. reported on a rapid loss of E‑cadherin and β‑catenin after 8 Gy total-body irradiation, which was associated with loss of mucosal barrier function in a rat model of irradiation-induced intestinal damage [36]. In summary, all in vivo studies yield a common conclusion of a rapid disruption of junction proteins after irradiation. The common denominator is irradiation doses equal to and higher than 4 Gy. A potential explanation for the discrepancy of the junction’s radiation response to either small or large fraction sizes might therefore be dose dependency, as also indicated by the recovery of junction proteins in the 2‑Gy dose group but not after 5 or 10 Gy in the in vitro study by De Carvalho et al.

While the physiological roles of E‑cadherin, occludin and claudin-1 are based on their functions within junctional complexes [12, 37], β‑catenin also exerts transcription factor activity as a key regulator of the canonical Wnt pathway. The Wnt/β-catenin axis plays a crucial role in tissue homeostasis, regeneration and repair [38], and was therefore investigated in a model of radiation-induced oral mucositis by Zhao et al., who reported on the mucoprotective potential of Wnt activation [39]. Without stimulation, β‑catenin expression, similarly to our results and in good agreement with other publications on normal mucosa [40, 41], remained membrane bound. Irradiation comprised one single high-dose fraction and Zhao et al. observed substantial membrane-bound β‑catenin deterioration, in line with other studies reporting junctional degeneration after single high-dose irradiation, as discussed above. Considering its twofold mechanism, the cellular localization of β‑catenin was focused on throughout our study period. No augmented nuclear translocation, hence activation of the Wnt/β-catenin signalling axis, was observed (data not shown). We therefore conclude that the Wnt/β-catenin signalling axis does not contribute to the radiation response of the oral epithelium to conventional fractionation doses and can be considered irrelevant for the interpretation of this study’s results.

The radiation-induced upregulation of epithelial cell contacts described in this study has, to our knowledge, not been demonstrated before and appears to be a new parameter of epithelial tolerance to fractionated irradiation. In our model, fractionated irradiation progressively increased the expression of tight as well as adherens junction proteins. In contrast to large single doses, conventional fractionation doses, as used in this study, seem to trigger responses other than cell death, i. e. changes in cell function. Similar findings have been reported for the central nervous system and skin [42–44]. We hypothesize that the upregulation of epithelial junctions potentially represents a twofold mechanism. The upregulation of adherens junctions appears to be a response to the abolished proliferation, thus immediately counteracting superficial cell loss via strengthening the anchorage of cells already present within the tissue at the time of irradiation. The upregulation of tight junctions might therefore indicate a response to the barrier deterioration. It is likely that both strengthening of tissue architecture and counteracting barrier loss are the basis of enhanced junction expression during fractionated irradiation. The effect is most pronounced from the second treatment week onwards, when repopulation is already fully active. Repopulation occurs in all early reacting normal tissues and translates into reduced radiation sensitivity with increased treatment time, thus more dose is necessary to achieve the same biological effect when fractionation rather than single-dose irradiation is administered. The characteristic epithelial radiation response is based on a profound proliferative reorganisation [45]. The newly discovered augmentation of cellular conjunction in response to fractionated irradiation, as presented in this study, could be an additional parameter of repopulation, with the loss in radiosensitivity being additionally based on augmented and reinforced epithelial cell conjunction.

Our findings highlight the importance of choosing a biologically relevant irradiation scheme for the radiation response being investigated. Clearly the response to single-dose irradiation is highly relevant for multiple scenarios, e. g. accidental exposure or stereotactic radiosurgery. However, the response to large single-dose irradiation does not necessarily reflect the same tissue’s response to clinically more relevant fractionation schemes.

## Conclusion

This study demonstrated a previously unknown response of junction proteins to fractionated irradiation with conventional daily doses. The expression of adherens junction marker proteins β‑catenin and E‑cadherin as well as the tight junction marker proteins claudin-1 and occludin rapidly increased during the course of 2 weeks of fractionation. The augmented expression was found in all epithelial layers and was highly significant for all proteins.

## Caption Electronic Supplementary Material

Mean Occludin and Claudin-1 expression values per animal, figure 3

Mean beta-catenin and e-cadherin expression values per animal, figure 2
